# Is easy-to-read easy for everyone? An eye-tracking study across the adult lifespan

**DOI:** 10.3389/fnagi.2026.1916339

**Published:** 2026-10-06

**Authors:** Sergio Navas-León, Jon Andoni Duñabeitia

**Affiliations:** Centro de Investigación Nebrija en Cognición (CINC), Department of Education, Universidad Nebrija, Madrid, Spain

**Keywords:** accessibility, adult lifespan, easy-to-read, eye tracking, older adults, reading, typography, visual processing

## Abstract

**Introduction:**

Easy-to-Read (E2R) formatting is widely recommended to improve reading accessibility, yet whether its typographic and structural features benefit visual processing and comprehension across the adult lifespan remains unclear.

**Methods:**

Using eye tracking, we examined whether E2R text formatting, relative to a standard control format, supports reading comprehension, reading time, and eye-movement behavior in 69 adults aged 18 to 83 years. Participants read short texts in both formats in a within-subject design while their eye movements were recorded, and reading comprehension was subsequently assessed. Data were analysed using mixed-effects models, with Text Format, Trial Order, Age, and theoretically relevant interactions entered as predictors across five dependent variables: comprehension accuracy, reading time, fixation count, average fixation duration, and average saccade amplitude.

**Results:**

E2R formatting did not significantly improve comprehension or reduce reading time relative to the control format. However, it was associated with shorter average fixation durations and larger average saccade amplitudes, suggesting some facilitation of visual processing during reading. Average fixation duration also showed a significant Text Format × Age interaction, whereby the difference between E2R and control formatting was more evident in younger adults and attenuated with increasing age, rather than becoming more pronounced among older readers as might be expected for an E2R format.

**Discussion:**

E2R formatting may facilitate some aspects of visual processing during reading across the adult lifespan, but these differences did not translate into improved comprehension or faster reading. Moreover, the observed visual-processing benefit did not become more pronounced with increasing age. These findings highlight the importance of evaluating the specific effects of E2R adaptations rather than assuming uniform benefits across adult age groups.

## 1 Introduction

Digital reading has become central to everyday life and to access to information, education, healthcare, public services, and civic participation (Eurostat, 2024; OECD, 2024). Yet reading in digital environments requires more than decoding text on a screen; it demands that users locate, evaluate, integrate, and process information within specific text, task, reader, and contextual conditions (Coiro, 2020; Salmerón et al., 2024; Van Moort et al., 2025). For this reason, the way written information is designed can either support or hinder access to digital content across different stages of adulthood, and accessibility is increasingly considered both a usability issue and a matter of equity and social participation (European Parliament and Council of the European Union, 2019; González-Sordé, 2025; Health Action International, 2024; United Nations, 2006).

In this context, Easy-to-Read (E2R) approaches have emerged as important frameworks for promoting accessible communication (González-Sordé and Matamala, 2024). E2R recommendations typically combine linguistic, structural, and visual principles, including the use of frequent words, simpler syntactic structures, clear organization, and accessible layout features such as left-aligned text, increased spacing, and reduced visual density (Deleanu et al., 2024). Although traditionally associated with people with intellectual disabilities, current conceptualizations define E2R as a resource for anyone experiencing barriers to understanding standard written information (Ekin et al., 2025; Schmutz et al., 2019), including barriers arising from literacy level, educational background, language experience, and, critically, normative ageing (Fuchs and Ohnuseit, 2025; OECD, 2024).

From the perspective of Cognitive Load Theory (Sweller, 1988), E2R formats may support reading by reducing unnecessary processing demands imposed by the presentation of information, thereby allowing readers to allocate more resources to comprehension and meaning integration. This account is also consistent with models of word recognition and lexical processing, as clearer layout, familiar vocabulary, simpler syntax, and supportive images may ease access to written meaning (e.g., Patterson and Shewell, 1987; Coltheart et al., 2001). However, empirical evidence on E2R effectiveness remains limited and heterogeneous (Cuitavi-Martín and Matamala, 2026; González-Sordé and Matamala, 2024; González-Sordé et al., 2025, 2026; Iacono, 2026; Sutherland and Isherwood, 2016; Wilson et al., 2026). For example, E2R adaptations may improve some outcomes, such as content recognition or task efficiency, but may also increase reading time or reduce subjective preference (Schmutz et al., 2016, 2019). This heterogeneity is particularly pronounced for visual and typographic features (e.g., spacing), which lack robust empirical support (Iacono, 2026). Given this limited evidence, E2R design choices, especially visual and typographic ones, should be understood as options requiring systematic empirical testing across outcomes and reader profiles (Navas-León and Duñabeitia, 2026).

Among these reading profiles, older adults deserve particular attention. Normative ageing is accompanied by ocular and neural changes that progressively affect visual processing, including reduced contrast sensitivity and diminished sensitivity to fine visual detail (Owsley, 2011, 2016). These changes increase susceptibility to visual crowding, the difficulty of identifying a letter or word when flanked by nearby visual elements (Whitney and Levi, 2011), making text harder to discriminate and rendering older readers more sensitive to typographic features that affect the clarity of written information (Chiu and Drieghe, 2023; Li et al., 2019). At the oculomotor level, older adults typically read more slowly, make more and longer fixations, and produce more regressions, while maintaining relatively preserved comprehension (Pagán et al., 2025). This pattern reflects a characteristic imbalance of cognitive ageing: fluid abilities such as processing speed, working memory, and executive control decline, whereas crystallized abilities, vocabulary and semantic knowledge, remain relatively stable (Gordon et al., 2016; Pegoraro et al., 2024), leading older adults to rely more heavily on linguistic knowledge and contextual information to sustain comprehension. Taken together, these perceptual and cognitive changes suggest that E2R features such as increased spacing, clearer layout, and reduced visual density could be particularly relevant for older adults (Fărcaşiu et al., 2023), by reducing the perceptual and cognitive load that densely formatted text imposes on a reading system whose resources are more narrowly allocated with age (Li et al., 2019).

In this context, a recent study by Navas-León and Duñabeitia (2026) examined young Spanish university students using three formats: a visually demanding hard-to-read format, a standard control format, and an E2R format with clearer layout, increased paragraph and word spacing, optimized contrast, and colour pictograms. The E2R format was associated with higher comprehension than the hard-to-read format, although not significantly different from the control format, and with shorter fixation durations and larger saccades than both comparison formats. These findings suggest that some combined visual and typographic E2R adaptations may support aspects of comprehension and visual processing in young adults without cognitive disabilities. Whether these benefits extend beyond young university students and across adulthood, however, remains an open question. This issue is particularly relevant in the European Union, where the median age of the population reached 44.9 years in 2025 and is projected to continue increasing in the coming decades; indeed, people aged 65 years and over already account for more than one fifth of the population, a proportion projected to continue rising (Eurostat, 2025). Against this demographic background, examining E2R effects may help clarify whether accessible text formats support reading across an increasingly age-diverse adult population.

The present study addresses this gap by examining whether text format, a control format versus an E2R format, affects reading comprehension, reading time, and eye-movement behavior across the adult lifespan. The E2R format manipulated several typographic variables commonly recommended in E2R guidelines, including text alignment, line spacing, inter-letter spacing, paragraph spacing, contrast, and visual support (Inclusion Europe, 2020; W3C Web Accessibility Initiative, 2023). This combined approach is consistent with ecological accounts of digital reading, which emphasize that typographic variables co-occur in naturalistic materials and should therefore be examined as integrated configurations rather than through isolated manipulations of single features (Scaltritti et al., 2019). This design can also be framed within the Simple View of Reading (Hoover and Gough, 1990), which separates decoding, that is, word reading, from linguistic comprehension and thereby supports treating word reading as a construct distinct from comprehension. The link between efficient word recognition and eye-movement control is instead specified by computational models such as the E-Z Reader model, in which fixation durations reflect the ease of ongoing lexical processing (Reichle and Drieghe, 2013). Accordingly, in the present study the eye-movement measures index online word-recognition efficiency and reading fluency, whereas the comprehension test captures offline, downstream comprehension. This distinction is relevant because online word-recognition efficiency has been shown to decline with age in spoken-language processing (Colby and McMurray, 2023); examining whether analogous age-related dynamics emerge during silent reading through eye-movement indices therefore represents a novel contribution of the present study.

To characterize eye-movement behavior, we focused on three global indices, each capturing a distinct process and motivating a specific directional prediction. Fixation count reflects the amount of visual sampling and processing devoted to the text, increasing with reading difficulty (Krejtz et al., 2016; Rayner et al., 2006). Average fixation duration indexes per-fixation processing load, with longer fixations reflecting slower cognitive processing (He et al., 2015; White et al., 2022). Average saccade amplitude captures the breadth of visual exploration and perceptual span, with longer saccades reflecting wider information extraction (Wang and Sparks, 2016).

Based on the evidence reviewed above, we formulated two sets of hypotheses. Regarding comprehension and reading time, we predicted that E2R formatting would reduce reading time and improve comprehension relative to the control format, that increasing age would be associated with longer reading times alongside relatively preserved comprehension, and that a Text Format × Age interaction would emerge such that the benefit of E2R would be more pronounced at the older end of the adult lifespan. Regarding eye-movement behavior, we predicted that E2R formatting would yield a more efficient oculomotor pattern, fewer fixations, shorter fixation durations, and larger saccade amplitudes, that increasing age would be associated with the opposite pattern, and that a Format × Age interaction would emerge such that E2R would attenuate age-related oculomotor costs more strongly among older adults.

## 2 Materials and methods

### 2.1 Participants

A total of 74 adults were initially recruited for the study. Before inclusion in the final sample, all participants underwent a screening procedure. This screening included the completion of the CAB PRO (CogniFit Inc., San Francisco, CA, USA) cognitive screening battery. Three participants were excluded because they did not complete the CAB PRO screening. Additionally, one participant was excluded because of missing reading comprehension questionnaire data, and one further participant was excluded after reporting medical comorbidities following study completion. This resulted in a final sample of 69 adults (*M* = 45.35, *SD* = 19.67; range = 18–83 years), of whom 50 were female. Recruitment took place between October 2025 and April 2026 through the laboratory’s internal platform, drawing participants from a pool of individuals registered to take part in experiments.

Inclusion criteria were: (a) normal or corrected-to-normal visual acuity, (b) being a native Spanish speaker, and (c) obtaining an age- and sex-adjusted score within the normal range on the Cognitive Assessment Battery (CAB) PRO, which provides a standardized score from 0 to 100. In the present sample, the mean CAB PRO score was 55.71 (SD = 18.91), and no participant was excluded based on this screening. Exclusion criteria included: (a) self-reported history of visual and ocular pathology, neurological disorders, or significant brain injury; (b) reported history of mental illness; (c) current use of recreational drugs; (d) incomplete data collection or unsuccessful eye tracking system calibration; and (e) non-native Spanish speakers.

Simulation-based model-aligned power analyses were conducted in R (R Core Team, 2024) to evaluate whether the sample size provided sufficient power to detect moderate expected Text Format effects within the mixed-effects modelling approach used for the five primary outcomes: reading comprehension, reading time, fixation count, average fixation duration, and average saccade amplitude. For the binomial comprehension model, power was estimated using the simr package (Green and MacLeod, 2016). For the linear mixed-effects models, robust Monte Carlo simulations were implemented using the fitted lme4 model objects (Bates et al., 2015). For each outcome-specific final model, power was estimated for the global effect of Text Format by comparing the fitted model with a reduced model that preserved the same random-effects structure but removed Text Format. This procedure incorporated the observed two-format within-subject design, the number of participants, the number of trials, the fixed-effect and random-effects structure of each final model, and the residual variance structure of each linear mixed-effects model. For reading comprehension, the dependent variable was binary at the item level, coded as 0 = incorrect and 1 = correct; therefore, a binomial mixed-effects model with a logit link was used. The simulation-based analysis indicated 95% CI [96.2, 98.3], with 69 participants. For the linear mixed-effects models, estimated power was 99.1% for reading time, 95% CI [98.3, 99.6]; 87.7% for fixation count, 95% CI [85.5, 89.7]; 100% for average fixation duration, 95% CI [99.6, 100]; and 100% for average saccade amplitude, 95% CI [99.6, 100]. Thus, the sample size provided adequate power to detect moderate expected Text Format effects across the outcomes.

The study adhered to the standards of the Declaration of Helsinki, and the procedure was approved by the ethics committee of Universidad Nebrija (approval code UNNE-2025-0118). All participants were informed about the experiment, given time to ask questions, and asked to sign informed consent forms. Participants received a monetary compensation (20 €) after participation.

### 2.2 Materials

Two fictional countries were created. Each country included 15 texts covering topics such as population and demographic trends, history, economy, politics, energy, education system, tourism, language, religion, cuisine, natural landscapes, healthcare system, infrastructure, climate, and geography, resulting in a total of 30 texts (see OSF project)^1^. The use of fictional rather than real countries minimized the influence of prior knowledge and allowed the two countries to be closely matched. Initial drafts of the texts were generated with the assistance of a large language model (GPT-5.5 Thinking, OpenAI) and were subsequently revised by the authors to ensure coherence, accessibility, and linguistic comparability across countries. During this process, complex sentences were simplified, jargon was reduced, and content was adapted for a broad audience. Simplification strategies included shortening lengthy sentences and minimizing the use of adverbs and superlatives. All materials were short informational, expository texts, descriptive in nature.

Furthermore, to ensure comparability across texts, lexical frequency and text-level characteristics were examined. Word frequency was controlled using EsPal (Duchon et al., 2013). No significant between-country differences were found in Zipf frequency values, *F*(1, 431) = 3.26, *p* = 0.072. No significant between-country differences were observed in the total number of words, *F*(1, 28) = 1.90, *p* = 0.179, lexical density, defined as the proportion of lexical content words relative to the total number of words, *F*(1, 28) = 0.01, *p* = 0.919, lexical diversity, defined as the proportion of unique lexical content words relative to the total number of lexical content words, *F*(1, 28) = 0.18, *p* = 0.674, or mean sentence length in words, *F*(1, 28) = 0.21, *p* = 0.651. Across the 30 texts, mean text length was 38.60 words (*SD* = 4.89, range = 30–49). Local referential cohesion, operationalized as the lexical overlap of content-word lemmas between adjacent sentences following the referential-cohesion approach of the Coh-Metrix framework (McNamara et al., 2014), also did not differ significantly between the two text sets, *F*(1, 28) = 2.39, *p* = 0.133.

The study used a within-subject design with two text formats: control, and E2R, such that each participant read texts in both formats. Four counterbalanced lists were used to rotate the assignment of text formats across countries, ensuring that each text appeared equally often in each format across participants.

In the control format (see Figure 1A), a sans-serif font (Lucida Sans) was used (font size = 18), with fully justified text, no paragraph breaks, 1.5-line spacing, default word spacing, and an adequate contrast ratio (background: #FFFFFF; font colour: #000000). In the E2R format (see Figure 1B), a sans-serif font (Lucida Sans) was used (font size = 18), with left-aligned text, 1.5-line spacing, increased paragraph spacing (twice the font size), and word spacing of at least 0.16 times the font size. An optimized contrast ratio was applied (background: #FFFFFF; font colour: #404040), and colour pictograms were included to support information processing.

**FIGURE 1:**
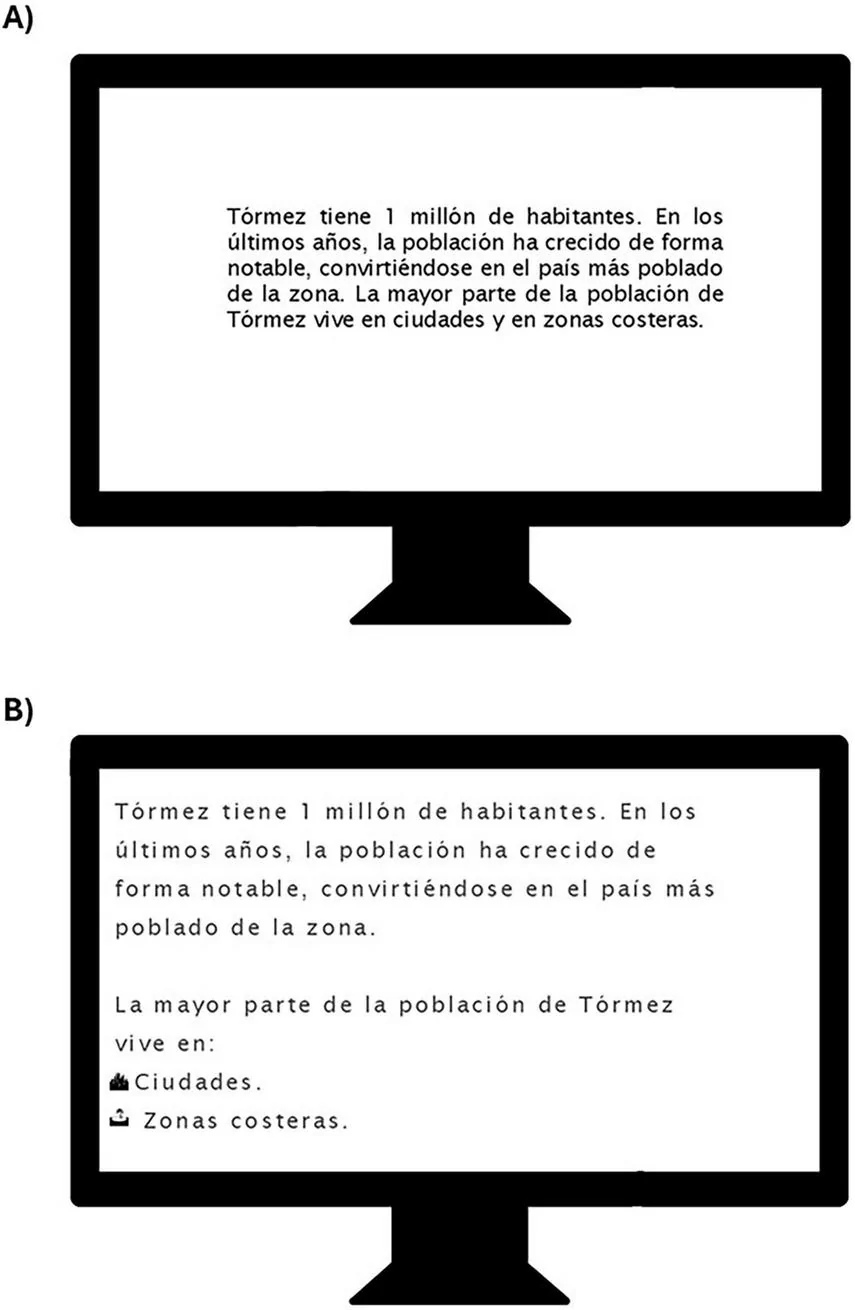
Examples of the two text formats: **(A)** control format, and **(B)** E2R format.

A multiple-choice questionnaire was designed to assess reading comprehension of the content associated with each country. The two questionnaires comprised 60 items in total (30 per country). The order of country presentations was counterbalanced across participants, and so was the order of questions and response options. Each question included four response options: one correct answer, two distractors based on information from other countries to increase task difficulty, and one option designed to assess plausibility and detect inconsistent or unrealistic choices. The full set of questionnaire items is available in the OSF project: https://osf.io/25ar8/overview.

### 2.3 Procedure

Participants were seated in a room with constant lighting and temperature. The experimental session lasted approximately 1 h. Before starting, participants were informed that the study concerned learning, that their participation was voluntary, and that they could withdraw at any time. Written informed consent was obtained prior to participation.

At the beginning of the experiment, participants provided basic sociodemographic information, including sex assigned at birth and age, and completed the cognitive assessment.

Later, participants were seated in front of the eye tracking system, facing a 27″, 165 Hz LED monitor (Lenovo Legion Y27h-30) with a resolution of 1920 × 1080 pixels. Eye movements were recorded using the EyeLink Portable Duo system (SR Research, Ontario, Canada), which tracked both eyes at a sampling rate of 500 Hz using the dark pupil–corneal reflection method. A height-adjustable chinrest was used to maintain head stability and a fixed viewing distance of 98 cm. A nine-point calibration and validation procedure was conducted individually for each participant. Calibration was accepted when the average validation error was below 0.5 ° of visual angle and the maximum error did not exceed 1.0°. When these criteria were not met, the calibration procedure was repeated. Drift correction was applied throughout the experiment.

Once calibrated, participants began the reading phase. They were instructed to read each text silently and advance to the next text by pressing the spacebar once they had finished reading. Before the experimental trials, participants completed five practice trials, which were used to familiarize them with the procedure and to resolve any questions. Once these practice trials had been completed, participants were presented with texts corresponding to different countries and text formats (control and E2R). Each trial began with a fixation cross presented at the center of the screen for 500 ms. This was followed by presentation of the text, which remained on screen for a maximum duration of 30,000 ms or until the participant pressed the spacebar to continue. Once a text was completed, it was no longer possible to return to it.

After completing the reading phase, participants were offered a 5-min break. They then performed a filler Flanker task (Eriksen and Eriksen, 1974), a cognitive control task in which they indicated the direction of a central arrow while ignoring surrounding distractor arrows. This task was included to minimize potential recency effects before reading comprehension questionnaires. The duration of the flanker task was identical for all participants (60,000 ms).

Participants then completed the two reading comprehension questionnaires (one per country), presented in counterbalanced order across participants, which together included 60 multiple-choice questions administered on a tablet device (Lenovo TB X306F). Each question was based on the texts read during the experiment and required participants to select the correct response from four options. Participants completed this phase at their own pace.

At the end of the experiment, participants were debriefed, provided with a summary information sheet, and thanked for their participation.

### 2.4 Data analysis

Dependent variables were reading comprehension, fixation count, average fixation duration, average saccade amplitude, and reading time, computed across the full text to capture global reading behavior, rather than being restricted to predefined AOIs. Reading comprehension was analysed as a binary outcome (correct vs. incorrect). Reading time was defined as the interval from text onset to keypress. Because the E2R adaptation required extensive layout editing, texts were presented as images, which precluded word-level interest areas and therefore first-fixation and gaze-duration measures. All indices were consequently extracted at the trial (whole-text) level using SR Research Data Viewer (SR Research Ltd., 2007), following the definitions in the EyeLink Data Viewer manual: average fixation duration was the mean duration of all fixations within each trial’s interest period, fixation count the total number of fixations, and average saccade amplitude the mean saccade amplitude across the trial. Average fixation duration therefore reflects per-fixation processing load across the whole text rather than initial word recognition specifically.

Data were pre-processed prior to modelling. Fixations were identified using the I-DT algorithm (minimum duration = 100 ms), and saccades were detected using velocity (30°/s) and acceleration (8000°/s^2^) thresholds, excluding saccades shorter than 2° (Holmqvist et al., 2011). Outliers were identified separately for each dependent variable within each participant × text format cell using a ± 2.5 SD criterion and recoded as missing values. Practice trials were excluded from the analysis.

Reading comprehension was analysed using a binomial generalized linear mixed-effects model (GLMM). Linear mixed-effects models (LMMs) were fitted for fixation count, average fixation duration, and average saccade amplitude, whereas reading time was analysed using an LMM fitted to log-transformed reading times. All models were specified within an *a priori* theoretically guided framework. Text Format was treated as the main experimental predictor, given that the primary aim of the study was to examine whether E2R formatting affected reading comprehension, reading time, and eye-movement behavior relative to the control format. Age was included as a continuous, mean-centered predictor because the study aimed to examine reading performance across the adult lifespan. The Text Format × Age interaction was therefore included. Trial Order was also considered to account for possible practice, fatigue, or order-related effects during the task.

Random-effect structures were specified to reflect the repeated-measures and crossed nature of the data. Random intercepts for Participant were prioritized because each participant contributed multiple observations, and random intercepts for Trial were prioritized because individual texts could differ in difficulty or visual processing demands. Following recommendations to account for both participant- and item-level variability and to consider random slopes when supported by the data (Barr et al., 2013), the candidate model set included random intercepts for Participant and Trial, random slopes for theoretically relevant within-subject predictors such as Text Format and Trial Order, and simpler random-effect structures derived from this maximal specification.

For each outcome, model comparison followed a sequential procedure. Models that did not converge or produced singular fits were first excluded. The remaining models were compared with their corresponding null models using likelihood-ratio tests, retaining only those that improved model fit. Model selection then favored parsimonious and interpretable models, with BIC used as the main criterion to balance model fit and complexity. Within this framework, models including the theoretically central predictors, particularly Text Format and Age, were prioritized when they were supported by model fit and did not introduce unnecessary complexity. Across outcomes, preference was also given to random-effect structures including random intercepts for both Participant and Trial, given their relevance for the experimental design.

Pairwise comparisons were conducted using estimated marginal means with Bonferroni correction when the retained model included Text Format as a predictor. Model diagnostics were performed using DHARMa residual diagnostics, including dispersion and zero-inflation tests where applicable.

Analyses were conducted in R version 4.5.3 (R Core Team, 2024). LMMs were fitted using lme4 (Bates et al., 2015), with lmerTest (Kuznetsova et al., 2017) used for inference in linear mixed models. Estimated marginal means and Bonferroni-adjusted pairwise comparisons were obtained with emmeans (Lenth et al., 2019), and model diagnostics were conducted using DHARMa (Hartig et al., 2024). Statistical significance was set at *p* < 0.05 for all analyses.

## 3 Results

Simulated residual analyses showed no evidence of dispersion or zero-inflation problems. Dispersion estimates were close to 1 and non-significant across reading comprehension, fixation count, average fixation duration, average saccade amplitude, and log-transformed reading time (ps ≥ 0.80), while zero-inflation tests were also non-significant (ps = 1). Participant-level descriptive statistics for all outcome variables by Text Format are presented in Table 1. Results of the mixed-effects models are reported below.

**TABLE 1:** Descriptive statistics (M and SD) for each outcome by text format, computed on participant-level means.

Outcome	Control M (SD)	E2R M (SD)
Reading comprehension (% correct)	72.8 (18.3)	74.3 (15.7)
Reading time (ms)	14,925 (4,850)	14,632 (4,923)
Fixation count	56.5 (17.3)	56.6 (18.2)
Average fixation duration (ms)	219 (32.0)	213 (29.7)
Average saccade amplitude (°)	3.07 (0.52)	3.18 (0.54)

### 3.1 Reading comprehension

The best-fitting model retained the fixed effects of Text Format, Trial Order, and Age, with a random intercept for Participant:


R⁢e⁢a⁢d⁢i⁢n⁢g⁢c⁢o⁢m⁢p⁢r⁢e⁢h⁢e⁢n⁢s⁢i⁢o⁢n∼T⁢e⁢x⁢t⁢F⁢o⁢r⁢m⁢a⁢t+T⁢r⁢i⁢a⁢l⁢O⁢r⁢d⁢e⁢r

   +Age+(1|Participant)

Raw participant-level accuracy was descriptively slightly higher in the E2R format (*M* = 74.3%, *SD* = 15.7) than in the Control format (*M* = 72.8%, *SD* = 18.3). The model yielded a marginal *R*^2^ of 0.010 and a conditional *R*^2^ of 0.206. Fixed-effect estimates are summarized in Table 2. Reading comprehension did not differ significantly between formats and was not significantly associated with Age. However, comprehension accuracy increased across Trial Order (see Figure 2A), indicating better performance as participants progressed through the task.

**TABLE 2:** Fixed-effects estimates from the mixed-effects models for each outcome.

Outcome	Predictor	β	SE	95% CI	*P*
Reading comprehension	Text format	−0.045	0.037	[−0.118, 0.028]	0.232
	Age	−0.136	0.116	[−0.363, 0.091]	0.245
	Trial order	0.147	0.038	[0.073, 0.221]	**< 0.001**
Reading time (log)	Text format	0.012	0.010	[−0.008, 0.031]	0.255
	Age	0.073	0.036	[0.003, 0.143]	**0.045**
	Trial order	−0.036	0.006	[−0.048, −0.023]	**< 0.001**
Fixation count	Text format	−0.072	0.545	[−1.142, 0.997]	0.895
	Age	3.631	2.020	[−0.329, 7.590]	0.077
	Trial order	−1.647	0.393	[−2.418, −0.876]	**< 0.001**
	Text format × trial order	0.931	0.310	[0.324, 1.539]	**0.003**
Average fixation duration	Text format	2.890	0.555	[1.802, 3.979]	**< 0.001**
	Age	2.160	3.653	[−5.00, 9.32]	0.556
	Trial order	−1.662	0.422	[−2.49, −0.83]	**< 0.001**
	Text format × age	−1.209	0.410	[−2.01, −0.41]	**0.003**
Average saccade amplitude	Text format	−0.055	0.016	[−0.085, −0.024]	**< 0.001**
	Age	−0.017	0.062	[−0.139, 0.105]	0.789
	Trial order	0.010	0.008	[−0.006, 0.027]	0.216

β, fixed-effect estimate; SE, standard error; CI, confidence interval. For reading comprehension, estimates are from a binomial mixed-effects model with a logit link and are expressed on the log-odds scale. Confidence intervals are Wald intervals. Significant effects (*p* < 0.05) are shown in bold.

**FIGURE 2:**
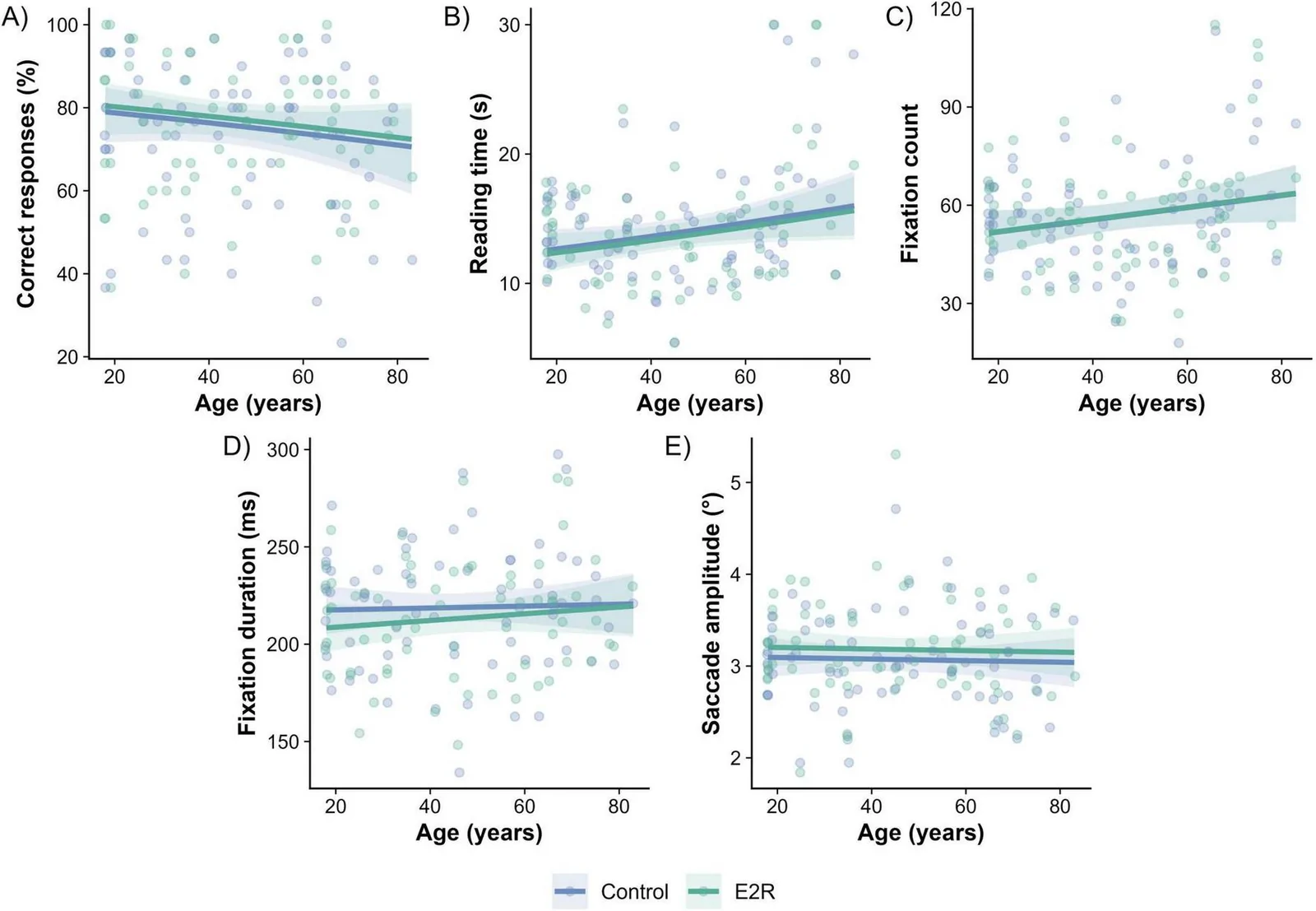
Comparison of behavioral and eye tracking outcomes across adult lifespan. Panels show the association between age and **(A)** correct responses in reading comprehension, **(B)** reading time, **(C)** average fixation count, **(D)** average fixation duration, and **(E)** average saccade amplitude for the Control and E2R formats. Points represent participant-level observed values for each format, aggregated across trials: percentage correct for reading comprehension and participant-level means for reading time and eye-movement measures. Solid lines represent model-based fitted trends, and shaded areas represent 95% confidence intervals.

### 3.2 Reading time

The best-fitting model retained the fixed effects of Text Format, Trial Order, and Age, with a random intercept for Participant and random intercepts and Trial Order slopes for Trial:


l⁢o⁢g⁢(R⁢e⁢a⁢d⁢i⁢n⁢g⁢T⁢i⁢m⁢e)∼T⁢e⁢x⁢t⁢F⁢o⁢r⁢m⁢a⁢t+T⁢r⁢i⁢a⁢l⁢O⁢r⁢d⁢e⁢r+A⁢g⁢e

+(1|Participant)+(1+TrialOrder||Trial)

Raw participant-level reading time was descriptively slightly higher in the Control format (*M* = 14,925 ms, *SD* = 4,850) than in the E2R format (*M* = 14,632 ms, *SD* = 4,923). The corresponding log-transformed means were *M* = 9.54 (*SD* = 0.31) for Control and *M* = 9.52 (*SD* = 0.32) for E2R. The model yielded a marginal *R*^2^ of 0.046 and a conditional *R*^2^ of 0.672. Fixed-effect estimates are summarized in Table 2. Reading time did not differ significantly between formats, indicating faster reading as participants progressed through the task, but decreased across Trial Order and increased with Age, indicating longer reading times at older ages (see Figure 2B).

### 3.3 Fixation count

The best-fitting model retained Text Format, Trial Order, Age, and the Text Format × Trial Order interaction, with random intercepts for Participant and Trial and a random Trial Order slope for Participant:


F⁢i⁢x⁢a⁢t⁢i⁢o⁢n⁢C⁢o⁢u⁢n⁢t∼T⁢e⁢x⁢t⁢F⁢o⁢r⁢m⁢a⁢t×T⁢r⁢i⁢a⁢l⁢O⁢r⁢d⁢e⁢r+A⁢g⁢e

+(1+TrialOrder||Participant)+(1|Trial)

Raw participant-level fixation count was similar across formats, with *M* = 56.5 (*SD* = 17.3) in the Control format and *M* = 56.6 (*SD* = 18.2) in the E2R format. The model yielded a marginal *R*^2^ of 0.034 and a conditional *R*^2^ of 0.630. Fixed-effect estimates are summarized in Table 2. There was no overall difference in fixation count between formats, and the effect of Age was not significant. Fixation count decreased across Trial Order, and this decrease differed significantly between formats (see Figure 2C).

Follow-up analyses showed a stronger decrease in fixation count across trials in the E2R format (slope = −2.58) than in the Control format (slope = −0.72), consistent with the significant Text Format × Trial Order interaction.

### 3.4 Average fixation duration

The best-fitting model retained Text Format, Age, Trial Order, and the Text Format × Age interaction, with random intercepts for Participant and Trial:


A⁢v⁢e⁢r⁢a⁢g⁢e⁢F⁢i⁢x⁢a⁢t⁢i⁢o⁢n⁢D⁢u⁢r⁢a⁢t⁢i⁢o⁢n∼T⁢e⁢x⁢t⁢F⁢o⁢r⁢m⁢a⁢t×A⁢g⁢e+

TrialOrder+(1|Participant)+(1|Trial)

Raw participant-level average fixation duration was lower in the E2R format (*M* = 213 ms, *SD* = 29.7) than in the Control format (*M* = 219 ms, *SD* = 32.0). The model yielded a marginal *R*^2^ of 0.014 and a conditional *R*^2^ of 0.734. Fixed-effect estimates are summarized in Table 2. Average fixation duration was significantly shorter in the E2R format than in the Control format and decreased across Trial Order, whereas the main effect of Age was not significant. Importantly, the effect of Text Format varied significantly with Age (see Figure 2D).

Follow-up analyses confirmed that the age-related slopes differed between formats (estimate = −2.42, *SE* = 0.82, *p* = 0.003), indicating that the E2R-related reduction in fixation duration was more pronounced at younger ages and attenuated with increasing age.

### 3.5 Average saccade amplitude

The best-fitting model retained the fixed effects of Text Format, Trial Order, and Age, with random intercepts for Participant and Trial:


A⁢v⁢e⁢r⁢a⁢g⁢e⁢S⁢a⁢c⁢c⁢a⁢d⁢e⁢A⁢m⁢p⁢l⁢i⁢t⁢u⁢d⁢e∼T⁢e⁢x⁢t⁢F⁢o⁢r⁢m⁢a⁢t+T⁢r⁢i⁢a⁢l⁢O⁢r⁢d⁢e⁢r

+Age+(1|Participant)+(1|Trial)

Raw participant-level average saccade amplitude was greater in the E2R format (*M* = 3.18°, *SD* = 0.54) than in the Control format (*M* = 3.07°, *SD* = 0.52). The model yielded a marginal *R*^2^ of 0.008 and a conditional *R*^2^ of 0.672. Fixed-effect estimates are summarized in Table 2. Average saccade amplitude was significantly larger in the E2R format than in the Control format, indicating longer eye movements during E2R reading, whereas neither Trial Order nor Age showed a significant effect (see Figure 2E).

## 4 Discussion

The present study aimed to examine whether E2R formatting, relative to a standard control format, supports reading comprehension, reading time, and eye-movement behavior across the adult lifespan. We hypothesized that E2R formatting would improve comprehension and reduce reading time, produce a more efficient oculomotor profile, fewer fixations, shorter fixation durations, and larger saccade amplitudes, and that these benefits would be more pronounced with increasing age. Overall, these hypotheses were only partially supported: E2R formatting produced clear benefits in fixation duration and saccade amplitude, but it did not significantly improve comprehension or reduce reading time, and the age-related moderation observed for fixation duration was opposite to the predicted direction.

### 4.1 Reading comprehension and reading time

The reading-comprehension hypothesis was not supported, as E2R formatting did not significantly improve comprehension relative to the control format, nor was there evidence that its effect varied as a function of age. Although comprehension was descriptively slightly higher in the E2R format, this difference was small and non-significant. This finding is consistent with a broader pattern in the E2R and Easy Language literature, where improvements in comprehension are not always observed (Ekin et al., 2025; González-Sordé et al., 2025; Rivero-Contreras et al., 2021). However, the considerable methodological heterogeneity across studies limits direct comparisons of results (González-Sordé and Matamala, 2024). More specifically, comprehension has been assessed very differently across studies, including free recall and recognition tasks, true/false judgements, open-ended questions, and multiple-choice questions with different numbers of response options and re-reading conditions (Schmutz et al., 2019; Rivero-Contreras et al., 2021; González-Sordé et al., 2025, 2026; Ekin et al., 2025).

The reading-time hypothesis was only partially supported, since age was associated with longer reading times, whereas E2R formatting did not significantly reduce reading time relative to the control format or attenuate age related slowing. The absence of a format effect on reading time aligns with findings from Schmutz et al. (2019), who reported that accessibility adaptations do not systematically reduce reading time in people without disabilities and may occasionally slow it down when additional visual or structural elements demand processing. The positive association between age and reading time is nevertheless consistent with well-established evidence on processing speed decline in ageing (Kliegl et al., 2004; Stine and Hindman, 1994; Zhang et al., 2022). It should be noted, however, that reading time was measured from text onset to keypress and may therefore include a small motor-execution component. To assess its influence, we examined the interval between the final fixation and the end of the interest period. This exploratory check showed no significant differences between text formats or association with age^2^, suggesting that motor-response timing does not substantially affect the interpretation of the reading-time findings.

### 4.2 Eye-movement behavior

The fixation-count hypothesis was not supported, as E2R formatting did not significantly reduce the number of fixations relative to the control format, and there was no clear evidence that fixation count was modulated by age in the predicted direction. Thus, any facilitation produced by E2R appears not to have operated through a reduction in the global amount of fixational sampling across the text.

The fixation-duration hypothesis was partially supported, because E2R formatting produced shorter average fixation durations than the control format, although the age-related moderation was opposite to the predicted direction. Shorter fixation durations are commonly interpreted as reduced processing demands per fixation unit (Liu et al., 2022), suggesting that E2R layout features may have facilitated fixation-level processing. More specifically, shorter fixation durations under E2R may index more efficient online word recognition and reading fluency (Rayner, 1998, 2009; Rayner et al., 2010; Reichle and Drieghe, 2013) rather than gains in offline comprehension, consistent with the distinction between decoding and linguistic comprehension drawn by the Simple View of Reading (Hoover and Gough, 1990). Because the present indices were computed at the whole-text level, we cannot fully disentangle word recognition and fluency from comprehension; we therefore advance this as a candidate explanation for the observed dissociation between eye-movement benefits and comprehension, and as a priority for future research using word-level measures. However, this benefit was more pronounced in younger adults and attenuated with increasing age, contrary to the prediction that older adults would benefit more from E2R formatting. One possible interpretation is that, in older adults, fixation behavior may be more strongly constrained by the coordination of multiple perceptual, attentional, oculomotor, and linguistic processes than by the visual clarity provided by E2R layout features, which appeared to be more evident in younger readers (Gordon et al., 2016; Pegoraro et al., 2024).

The saccade-amplitude hypothesis was partially supported, as E2R formatting produced larger average saccade amplitudes than the control format, although this effect was not significantly moderated by age. Larger saccades suggest that readers extracted information from a wider perceptual span, consistent with more efficient visuospatial processing (Chiu and Drieghe, 2023). This finding is theoretically consistent with accounts of visual crowding and spacing effects in reading, as increased spacing may reduce lateral interference between adjacent letters and words and support more efficient forward eye movements (Chiu and Drieghe, 2023). Consistent with this account, adjacent words have been shown to interfere with visual word recognition, with interference increasing at closer inter-word distances (Zelihic et al., 2026). The present combination of shorter fixation durations and larger saccade amplitudes is broadly compatible with the idea that E2R formatting may facilitate more efficient visual processing. Nevertheless, more efficient oculomotor patterns, such as shorter fixations and larger saccades, do not necessarily reflect faster or deeper processing, as they may also arise from lower engagement or reduced comprehension (Mézière et al., 2026).

### 4.3 Limitations and future directions

Several limitations should be acknowledged. First, the E2R manipulation combined multiple typographic and visual features, including alignment, spacing, contrast, and pictorial support. Although this reflects the ecological nature of accessible digital texts (Scaltritti et al., 2019), it prevents us from identifying which specific feature drove the observed effects. In addition, because texts were presented as images to preserve the E2R layout, eye-movement indices were computed at the whole-text level; the absence of word-level measures, such as first-fixation or gaze duration, prevents us from isolating word-recognition and reading-fluency processes from downstream comprehension, an important direction for future research (see section “4.2 Eye-movement behavior”). Future studies should isolate individual components, particularly given the heterogeneous evidence for typographic features (e.g., spacing) (Iacono, 2026). Second, the sample was cognitively healthy and may not fully represent older adults with lower educational background, reduced digital literacy, or stronger age-related perceptual difficulties. This may have attenuated potential E2R effects, especially for comprehension. Finally, the study focused on performance and eye-movement outcomes but did not assess subjective experience, perceived difficulty, or format preference, which may diverge from objective performance (Schmutz et al., 2019). While the multiple-choice comprehension items were not pre-tested prior to the study, a *post-hoc* analysis restricted to the Control condition showed comparable mean item difficulty across countries and no excessively difficult items^3^. Relatedly, the reliance on a multiple-choice comprehension measure may have shaped the results, as different assessment formats, such as recall versus recognition, impose different retrieval demands and can yield divergent outcomes (Cao and Kim, 2021; Ozuru et al., 2013); future studies should compare E2R effects across comprehension measures.

## 5 Conclusion

The present study provides evidence that E2R formatting was associated with oculomotor benefits, specifically shorter fixation durations and larger saccade amplitudes, in adults across a wide age range. These findings are consistent with the view that typographic accessibility features can reduce perceptual and attentional demands during reading. However, these benefits did not translate into significant improvements in comprehension or overall reading time. These findings contribute to a growing body of empirical evidence suggesting that E2R adaptations can confer measurable benefits beyond populations with intellectual disabilities, while also highlighting that such benefits are not uniform across the adult lifespan or across different reading outcomes. From a practical standpoint, the results support the use of E2R layout features in digital communication aimed at broad audiences but underscore the need for outcome-specific and population-specific testing before assuming that accessibility improvements will generalize uniformly.

## Data Availability

The datasets presented in this study can be found in online repositories. The names of the repository/repositories and accession number(s) can be found in the article/supplementary material.

## Footnotes

1
https://osf.io/25ar8/overview

2 Post-fixation response latency was similar across text formats (Control: *M* = 270.08 ms; E2R: *M* = 241.86 ms; *d* = 0.039; ps ≥ 0.370) and was not meaningfully associated with age (|r| ≤ 0.045; ps ≥ 0.776).

3 Tórmez: *M* = 0.72, *SD* = 0.13; Norvia: *M* = 0.73, *SD* = 0.14), with no significant difference in mean item difficulty, Welch’s *t*(57.81) = −0.37, *p* = 0.713, *d* = −0.10.
